# Prevalence of TB medication non-adherence and associated factors among adults attending a Regional Referral Hospital in Dar es Salaam, Tanzania

**DOI:** 10.1186/s12879-026-12886-7

**Published:** 2026-08-17

**Authors:** Tresphory Zumba, Candida Moshiro, Sabina Mugusi, Lulu Fundikira, Hussein H. Mwanga, Maryam Amour

**Affiliations:** 1 Department of Community Health, School of Public Health and Social Sciences, Muhimbili University of Health and Allied Sciences, Dar es Salaam, Tanzania; 2 Department of Epidemiology and Biostatistics, School of Public Health and Social Sciences, Muhimbili University of Health and Allied Sciences, Dar es Salaam, Tanzania; 3 Department of Clinical Pharmacology, School of Biomedical Sciences, Muhimbili University of Health and Allied Sciences, Dar es Salaam, Tanzania; 4 Department of Radiology, School of Clinical Medicine, Muhimbili University of Health and Allied Sciences, Dar es Salaam, Tanzania; 5 Department of Environmental and Occupational Health, School of Public Health and Social Sciences, Muhimbili University of Health and Allied Sciences, Dar es Salaam, Tanzania; 6 Ifakara Health Institute, Plot 463 Kiko Avenue Mikocheni, P.O. Box 78 373, Dar es Salaam, Tanzania

**Keywords:** Non-adherence to TB medication, Tuberculosis

## Abstract

**Background:**

Non-adherence to tuberculosis (TB) medication is a significant public health problem, associated with poor treatment outcomes such as drug resistance, relapse, increased morbidity and mortality. Ensuring adherence to TB medication is key for treatment success. To effectively promote adherence, it is important to have a clear understanding of the factors associated with non-adherence.

**Objective:**

The study aimed to determine the prevalence and factors associated with non-adherence to medications among TB patients attending a Regional Referral Hospital in Dar es Salaam.

**Methodology:**

A cross-sectional study was conducted at the TB clinic of this hospital. A total of 362 participants were selected using a systematic random sampling method. Data were collected through face-to-face interviews after obtaining informed consent. Non-adherence was assessed based on responses to four questions addressing forgetfulness, intentional breaks and recent missed doses. Participants answering “No” to all questions were classified as adherent; a “Yes” to any indicated non-adherence. Factors independently associated with non-adherence were determined using multivariable modified Poisson regression analysis.

**Results:**

The median age of study participants was 38 years (interquartile range: 30–46 years). The majority, 267 (73.8%) were aged between 18 and 45 years. Most participants, 246 (68%), were male. The prevalence of non-adherence to TB medications was 14.4%. In the multivariable modified Poisson regression model, non-adherence to TB medications was positively associated with methadone use (adjusted prevalence ratio (aPR) = 3.04; 95% CI: 1.69–5.46), spending 1–2 h at the hospital (aPR = 4.44; 95% CI: 1.16–16.97) and reporting a strong healthcare worker-patient relationship (aPR = 2.55; 95% CI: 1.56–4.17).

**Conclusions:**

Non-adherence to TB medication was relatively low compared to previous reports from sub-Saharan Africa (18.4% – 30.5%). Methadone use, shorter clinic visits duration and a very good relationship with HCWs were significantly associated with non-adherence to TB medications. These findings highlight the need to integrate substance use services with TB care and to explore how healthcare interactions and clinic processes influence treatment adherence.

**Clinical trial number:**

Not applicable.

**Supplementary Information:**

The online version contains supplementary material available at https://doi.org/10.1186/s12879-026-12886-7.

## Background

Tuberculosis (TB) is the 13th leading cause of death and the second leading infectious killer worldwide, after COVID-19 [1]. Majority of people affected by TB live in low- and middle-income countries [1]. In 2020, approximately 1.5 million people died from TB disease globally [1]. In 2022, about 10 million people worldwide, fell ill with TB, including 5.6 million men, 3.3 million women, and 1.1 million children [1]. According to a 2023 report by the World Health Organization (WHO), Democratic Republic of the Congo and Nigeria were the most affected countries in Africa [2] A 2022 report from the Centers for Disease Control and Prevention (CDC) indicated that in Tanzania, about 36% of TB patients were undiagnosed and untreated due to stigma and discrimination within the society [3].

Tuberculosis treatment necessitates six consecutive months of uninterrupted medication to ensure effectiveness. TB medications play a critical role in reducing disease transmission, morbidity, mortality, and disability [4]. It has been reported that approximately 85% of the patients who develop TB disease can be completely cured by using TB medications for six months [5]. To optimize TB treatment outcomes and enhance medication adherence, thereby preventing drug resistance, treatment failure, and relapse, the WHO introduced the directly observed treatment (DOT) strategy in 1998 in order to ensure effective treatment, follow-up, and monitoring of patients [4, 6]. Also, in order to improve medication adherence for TB patients, Patient Centered TB Treatment (PCT) was introduced in Tanzania in 2006. As a result, more than 75% of TB patients are now monitored and supervised at the community level. Individuals undergoing TB treatment are afforded the choice of DOT administered by healthcare professionals within a clinical setting or by trained non-medical personnel within their respective communities [7].

However, it has been shown from a study done in Dar es Salaam in 2016 that, patients on home-based DOTS were more likely to experience poor treatment outcomes and higher mortality rates, despite demonstrating good adherence to treatment [8]. In contrast, patients supervised through facility-based DOTS were less likely to die [8]. However, DOT and PCT methods are not currently used due to programmatic changes in the national TB control strategy, which may impact adherence to medications for TB patients [4]. Instead, TB patients are currently managed through routine clinic-based follow-up and self-administered therapy, supported by health education and periodic supervision by healthcare workers.

According to WHO, medication adherence refers to “the extent to which a person’s behavior-taking medication, following a diet, or executing lifestyle changes, corresponds with agreed recommendations from health care provider” [9]. Adherence to TB medication is crucial for treatment success, while non-adherence is associated with adverse outcomes such as increased mortality, morbidity and relapse [10]. Non-adherence to TB medications remains a significant obstacle facing TB control programs, hindering both prevention and treatment efforts. Patients who take less than 95% of their prescribed TB medications are considered non-adherent [11]. Poor medication adherence has a clear link with multi-drug resistant tuberculosis (MDR-TB) and about 28% of all MDR-TB cases in Tanzania come from Dar es Salaam region [12]. Apart from that, if TB is not well treated and controlled, it imposes a considerable economic burden on individuals and the community. A study conducted in Dar es Salaam revealed over 53% of TB patients borrowed money, some even sold personal assets, to afford expenses related to diagnosis, transportation, nutritional support, and other indirect costs, despite anti-TB medications being provided free of charge [13].

Adherence to tuberculosis medication is influenced by a complex interplay of factors, including patient-related issues such as alcohol consumption and side effects, as well as health system challenges, particularly prevalent in Sub-Saharan Africa [14–16]. Although TB medications are provided free of charge in Tanzania, the country continues to have a high TB burden, further complicated by HIV co-infection [13, 17]. The national non-adherence rate is 16.9%, highlighting the need for strengthened strategies to improve diagnosis, patient management, and education on medication adherence in order to achieve the global goal of ending the TB epidemic by 2030 [16]. While existing research has explored contributing factors, a clearer understanding of the specific determinants of non-adherence within the local context of a Regional Referral Hospital is essential. Therefore, this study aimed to determine the prevalence and factors associated with non-adherence to TB medications among patients receiving care at this facility.

## Methodology

### Study design

This study employed an analytical cross-sectional design, utilizing quantitative methods to collect and analyze data.

### Study setting

The study was conducted at a Regional Referral Hospital (RRH) in Dar es Salaam, Tanzania. This government-owned tertiary facility serves a predominantly urban low- and middle-income population and provides referral services to over 200 health facilities. It serves a catchment population of more than 2.2 million people and offers comprehensive TB services under the national DOTS program, alongside integrated care for HIV and substance use disorders [17].

### Study population

The study population comprised adult TB patients (aged 18 years and above) who were diagnosed and receiving treatment at the hospital’s TB clinic. Eligible participants were those on the standard six-month TB treatment regimen and refilling their medications at the outpatient TB clinic. Patients in very poor general condition who were unable to participate in the interviews were excluded.

### Sample size

Sample size was calculated using the formula for estimating single proportion:

$$\:\mathrm{n}=\frac{{\mathrm{z}}^{2}\mathrm{p}(100-\mathrm{p})}{{\varepsilon}^{2}}$$

Where: -

n = sample size

z= standard normal deviate set at 1.96 for 95% confidence interval

ε = margin of error, set at 5%

P=expected prevalence of non-adherence to TB medication (30.5%), obtained from a previous study [18].

Substituting these values:

$$\:\mathrm{n}\:=\:\frac{{\left(1.96\right)}^{2}\:\mathrm{x}\:30.5\:\mathrm{x}\:(100\hspace{0.17em}-\hspace{0.17em}30.5)}{\left({5}^{2}\right)}\hspace{0.17em}=\hspace{0.17em}326$$

After adding 10% to account for potential non-response, the final sample size was adjusted to: *n* = 326/0.9 = 362.

Therefore, a total of 362 participants were recruited for this study.

### Sampling strategy

This study targeted adult patients with TB who are attending clinic for medication refills. These refill sessions are usually conducted twice a week, with a high daily attendance ranging from 50 to 100 patients. Based on this volume, we estimated a total flow of approximately 1,500 patient visits (N) over the 8-week data collection period (about 16 clinic days). To ensure every patient had an equal probability of selection, we applied systematic random sampling. The sampling interval (k) was calculated using the formula:

$$\:\mathrm{k}\hspace{0.17em}=\hspace{0.17em}\mathrm{N}/\mathrm{n}$$

where *N* = 1,500 (total population) and *n* = 362 (sample size), yielding *k* ≈ 4.14, which was approximated to 4. The first participant was selected randomly from the clinic register, and subsequent participants were chosen after every fourth entry until the required sample size of 362 was reached.

### Variables and measurements

#### Dependent variable

Non-adherence to TB medications: Defined as a patient’s inability or refusal to take TB drugs as prescribed. It was measured using a 4-item structured questionnaire with “yes” or “no” responses as following:

1. Do you sometimes find it difficult to remember to take your medication?
2. When you feel better, do you sometimes take a break from your medication?
3. Thinking back over the past four days, have you missed any doses?
4. Sometimes if you feel worse when taking the medicine, do you stop taking it?

Participants answering “yes” to at least one question were classified as non-adherent.

#### Independent variables

Care-seeking from traditional healers: Defined as consulting unregulated, independent health providers or using herbal medications. This was measured by face-to-face interviews asking about participants’ behavior regarding traditional medicine use.

Alcohol consumption: Measured using the WHO Alcohol Use Disorder Identification Test (AUDIT), a 10-item questionnaire scored 0–4 per item. Total scores were categorized as low risk [1–7], harmful consumption [8–14], and likely alcohol dependence (≥ 15).

Waiting time: Defined as the duration from arrival at the TB clinic to leaving with prescribed medication. Was measured by participant self-report during interviews.

Tobacco smoking: Use of cigarettes, snuff, or chewing tobacco. Measured by interview and categorized as smoker or non-smoker using a 1–4 scale.

Medication side effects: Self-reported adverse reactions after taking TB medications, recorded as yes/no.

Health education: Receipt of health information or counseling from healthcare workers during TB clinic visits, measured as yes/no.

Comorbidities: Presence of additional chronic or long-term diseases, assessed via self-report and review of attendance at other clinics.

Education level: Highest level of formal education completed, measured on a 1–7 scale.

Patient–healthcare provider relationship: Participants’ perception of healthcare workers’ attitude, caring, and behavior, measured on a 1–5 Likert scale.

Age: This is the year from birth to present and was measured in years.

Sex: This is the defined as the physical appearance of either being male or female. This was measured by self-reported as male or female.

Pill burden: Number of tablets taken regularly; assessed by asking whether participants tolerated TB medications without any problem.

Occupation: Employment status, measured on a 1–5 scale (unemployed, self-employed, government employed, private employed, or previously employed but stopped due to TB).

Health facility distance: Time spent traveling to the nearest health facility, measured in minutes.

Family support: Assistance provided by family members regarding treatment information, emotional support, and financial help. Rated as never, sometimes, or always.

### Data collection methods

Data were collected at the TB clinic of the RRH for a period of 8 weeks, from March to April 2023. Information was collected simultaneously from each participant to investigate the association between exposure to risk factors and the outcomes of interest. Participants selection was random. The interview was pretested on 20 TB participants at another RRH, with similar characteristics as the index hospital, to ensure the validity and reliability. Feedback from the pretest was incorporated to improve the questionnaire [19]. Following their clinic visit, participants were invited to a private room for a face-to-face interview, conducted with N95 mask protection. Participants were invited to take part in the study after being seen by the health care workers at the TB clinic. Before the interview, fieldworkers provided a study briefing, and voluntary verbal and written informed consent was obtained from each participant. Informed consent was obtained through signature for literate participants, and with the aid of an impartial witness for those unable to read or write. Each interview lasted 20–30 min.

### Data analysis

Data were checked for completeness, coded and entered into Microsoft Excel, then exported to STATA software version 14.0 for analysis. Descriptive analysis was used to describe the sample characteristics. The prevalence of non-adherence to TB medication was calculated as the proportion of patients who were non-adherent out of the total sample. Medians and inter-quartile ranges were used to summarize continuous variables.

Since the outcome variable (non-adherence) was relatively common, conventional logistic regression was not used, as it tends to overestimate odds ratios in such cases. Instead, a modified Poisson regression model with robust standard errors was employed to directly estimate prevalence ratios (PRs). This approach is suitable for binary outcomes and provides a more accurate measure of association for non-rare events.

Variables with a *p* ≤ 0.2 in bivariate analysis were included in the multivariable modified Poisson regression model with robust standard errors to control for potential cofounders. Crude prevalence ratio (cPR), adjusted prevalence ratio (aPR) and 95% confidence intervals (CI) were calculated. We considered results statistically significant if the *p*-value obtained was < 0.05.

## Results

### Socio-demographic characteristics

We interviewed a total of 362 participants. The median age was 38 years (interquartile range: 30–46 years) (Table 1). The majority, 267 (73.8%) of the participants were aged between 18 and 45 years. Of the participants, 246 (68%) were male and 186 (51.4%) were married. Most participants, 155 (42.8%), had a primary education, and nearly half, 182 (49.7%) of the participants were unemployed.

**Table 1:** Socio-demographic characteristics of the study participants (*n* = 362)

Variable	Frequency	Percent
Age (years)		
18–45	267	73.8
46–60	85	23.4
> 60	10	2.8
Median age in years (Interquartile range)	38 (30, 46)	
Sex		
Male	246	68.0
Female	116	32.0
Marital status		
Single	128	35.3
Married	186	51.4
Divorced or widowed	48	13.3
Level of education		
No formal	19	5.3
Primary	175	48.3
Secondary and above	168	46.4
Occupation		
Unemployed	182	49.7
Employed	180	50.3
Distance to health facility (minutes)		
< 30	145	40.1
≥ 30	217	59.9

Table 2 Presents the behavioral characteristics of the study participants. The majority, 283 (78.1%), reported not consuming alcohol, while 10 (2.8%) were alcohol dependent. Most participants, 309 (85.4%), were non-smokers.

### Behavioral characteristics of study participants

**Table 2:** Behavioral characteristics of the study participants (*n* = 362)

Variable	Frequency (*n*)	Percent (%)
Alcohol consumption		
Not consuming	283	78.1
Risky consumption	4	1.1
Harmful consumption	65	18.0
Alcohol dependent	10	2.8
Cigarette smoking		
Yes	53	14.6
No	309	85.4

### Health system and support characteristics

Table 3 presents the health system and social support characteristics of the study participants.

The majority, 220 (60.8%), reported always receiving family support, while 37 (10.2%) were using methadone. Most participants, 246 (68.0%) spent less than one hour at the hospital, and the majority, 250 (69.1%) described their relationship with healthcare workers as “very bad to good.”

**Table 3:** Health system and support characteristics of study participants (*n* = 362)

Variable	Frequency (*n*)	Percent (%)
Methadone use		
Yes	37	10.2
No	325	89.8
Family support		
Always	220	60.8
Somehow	97	26.8
Never	45	12.4
Relationship with HCWs		
Very good	112	30.9
Very bad to Good	250	69.1
Time spent at the hospital (hours)		
< 1	246	68.0
1–2	77	21.3
≥ 3	39	10.7

The overall prevalence of non-adherence to TB medications found in this study was 14.4%, based on self-reported responses from participants (95% CI: 11.1%-18.4%).

### Factors associated with non-adherence to TB medications

Univariable analysis showed a higher prevalence of non-adherence to TB medication among participants aged 46–60 years (cPR = 1.71;95% CI: 1.02–2.88) and those with no formal to primary level of education (cPR = 1.95; 95% CI: 1.12–3.38) (Table 4). There was no association between non-adherence to TB medications and participants’ sex.

Multivariable modified Poisson regression model identified three factors that were independently associated with non-adherence to TB medications. Higher prevalence of non-adherence was observed among participants who reported methadone use (aPR 3.04; 95% CI: 1.69–5.46), those who spent 1–2 h at the hospital during clinic visits (aPR = 4.44; 95% CI, 1.16–16.97) and those who reported a very good relationship with HCWs (aPR = 2.55; 95% CI: 1.56–4.17).

**Table 4:** Multivariable analysis of the factors associated with non-adherence to TB medication

Variable	Total	Univariable analysis			Multivariable analysis		
		cPR	95% CI	*P* – value	aPR	95% CI	*P* - value
Age (years)							
18–45	267	Ref			Ref		
46–60	85	1.71	1.02–2.88	0.042	1.12	0.63–1.99	0.710
> 60	10	0.81	0.12–5.34	0.826	0.47	0.04–5.55	0.551
Sex							
Male	246	1.76	0.94–3.29	0.078	1.22	0.63–2.40	0.556
Female	116	Ref			Ref		
Level of education							
No formal to primary	194	1.95	1.12–3.38	0.018	1.74	0.96–3.16	0.069
Secondary and above	168	Ref			Ref		
Alcohol consumption							
Yes	79	0.38	0.16–0.93	0.330	0.54	0.24–1.23	0.142
No	283	Ref			Ref		
Family support							
Never	45	1.00	0.91–1.09	0.961	0.71	0.31–1.63	0.421
Somehow	97	1.10	1.02–1.19	0.015	1.41	0.81–2.47	0.228
Always	220	Ref			Ref		
Relation with HCWs							
Very good	112	2.07	1.26–3.40	0.004	2.55	1.56–4.17	0.001
Very bad to Good	250	Ref			Ref		
Methadone							
Yes	37	3.90	2.41–6.31	< 0.001	3.04	1.69–5.46	0.001
No	325	Ref			Ref		
Time spent at hospital (hours)							
< 1	246	2.54	0.63 − 10.16	0.189	2.48	0.69–8.91	0.163
1–2	77	4.56	1.11 − 18.66	0.035	4.44	1.16–16.97	0.029
≥ 3	39	Ref			Ref		

Key: aPR: Adjusted Prevalence Ratio, Ref=Reference Category, HCW = Health Care Workers

## Discussion

This study aimed to determine the prevalence and factors associated with non-adherence to TB medications among adult patients attending a Regional Referral Hospital in Dar es Salaam. The prevalence of non-adherence in this study was 14.4%. Factors independently associated with non-adherence included methadone use, spending one to two hours at the health facility, and having a very good healthcare worker–patient relationship.

The 14.4% prevalence of non-adherence to TB medications observed in our study is consistent with findings from other studies conducted elsewhere. For instance, the finding is similar to that found in the study conducted in Kosovo in 2017 which reported a non-adherence of 14.5% [20], and is also close to the 18.4% found in a 2020 study from Ghana [4]. A systematic review and meta-analysis by Zegeye et al. reported a slightly higher prevalence of 21.29% [21]. In contrast, the studies from South Korea and Nigeria reported substantially higher rates of non-adherence: 45% and 30.5%, respectively [22, 23]. These discrepancies could be attributed to several factors, including differences in study populations, sampling techniques, geographical settings, and sample sizes. Furthermore, as our study was conducted at a single referral hospital, direct comparability with multi-site or national studies may be limited. The relatively lower prevalence observed in our setting could also reflect strengthened patient–provider relationships and ongoing national TB control efforts under Tanzania’s National Strategic Plan VI, which have contributed to reductions in TB incidence and mortality [10].

The study revealed an association between methadone use and non-adherence to TB medication, with methadone users demonstrating higher prevalence of non-adherence. In contrast, a study conducted in Ukraine found that TB patients receiving methadone maintenance treatment (MMT) had better adherence to TB medication compared to those not on MMT. This improved adherence was attributed to increased retention in treatment when MMT was integrated with TB care among people who inject drugs [24]. Similarly, findings from a cross-sectional survey conducted in Kenya by the National Tuberculosis, Leprosy and Lung Disease Program in 2018 indicated that participants with substance use behavior (not specifically substitution therapy) were more likely to adhere to TB medications compared to non-users [25]. The lower adherence observed in our study among methadone users may be due to several interrelated factors. First, the comorbidity of TB and substance use disorder often results in more complex treatment regimens, which can be challenging to manage. Many methadone users also experience homelessness, limiting their ability to maintain regular medication routines and access consistent healthcare [26]. Psychiatric comorbidities, such as depression and anxiety, may also contribute to non-adherence by impairing memory and increasing the likelihood of missed clinic visits. Additionally, patients may fear potential side effects from taking both TB medications and methadone, which can lead to dose reduction or discontinuation. Logistical challenges may further complicate adherence. For example, patients who must attend both TB and methadone clinics on the same day may prioritize methadone treatment, leading to missed TB appointments due to time constraints. Stigma and discrimination related to substance use disorder can also affect adherence, as patients may experience shame or self-blame, which undermines motivation to follow treatment plans. Furthermore, ongoing substance use while on methadone treatment may lead to relapse, causing individuals to prioritize obtaining illicit drugs over adhering to prescribed medication regimens [27]. To address these challenges, a multidisciplinary care approach is essential. Evidence from Ukraine shows that integrating methadone maintenance treatment with TB services within the same facility improves treatment retention and adherence among people who inject drugs [24]. Similarly, experiences from Kenya emphasize the importance of tailored adherence support for patients with substance use behaviors [25]. These examples demonstrate that integrated service delivery and harm reduction strategies can facilitate effective treatment of both conditions and enhance TB medication adherence among methadone users.

Findings from our study showed that participants who reported having a very bad to good relationship with their healthcare workers (HCWs) were more likely to adhere to TB medication compared to those who reported a very good relationship. Similarly, findings from a 2025 study in Uganda reported that very good HCWs-patient relationships reflected by positive coefficients for medication reminders and emotional support were paradoxically associated with non-adherence to TB medications [28]. Contrary to the findings, a study from Kenya found that participants who perceived friendly HCWs had higher rates of adherence than those who described their HCWs as unfriendly [29]. Interestingly, a study from Ethiopia found no statistically significant association between the quality of patient**-**HCWs relationships and TB medication adherence [30]. A very good patient-HCWs relationship may contribute to non-adherence if patients become overly reliant on the HCWs, perceiving less personal responsibility for their medication. This is more likely when the HCWs is seen as consistently available and supportive, leading to reduced patient accountability and potentially contributing to non-adherence [28]. Another possible explanation is the emergence of emotional or social complexities when the patient-HCWs relationship becomes too personal, potentially blurring professional boundaries and allowing treatment decisions to be influenced by non-medical factors such as shame or emotional involvement, which may contribute to non-adherence to TB medications.

Interestingly, findings from the current study indicate that participants who spent a shorter amount of time (1–2 h) at the hospital seeking healthcare services were more likely to be non-adherent to TB medications compared to those who spent a longer time (≥ 3 h). This result contrasts with findings from several studies conducted globally. For instance, a systematic review and meta-analysis conducted in 2019 by Zegeye et al. found that patients who experienced longer waiting times (≥ 1 h) were more likely to be non-adherent to TB medications compared to those with shorter waiting times [21]. Similarly, a 2020 study from Nepal by Baral et al. reported that longer waiting times were associated with higher levels of non-adherence to TB treatment [31]. A possible explanation for the differing findings in the present study could be that patients who spend more time at the facility may receive more comprehensive attention, including engagement in peer health education and counseling. In contrast, those seen for shorter time or more quickly may receive rushed or superficial care, potentially reducing their trust in healthcare providers and their willingness to follow treatment instructions. This rushed interaction might lead patients to underestimate the seriousness of their condition, which can reduce motivation and lead to non-adherence. Supporting this view, a systematic review study in 2016 by Michael et al. highlighted that spending more time in patient-provider communication helps to build trust and positively influences adherence to TB treatment [32]. Likewise, a 2012 study by James et al. found that one-on-one or group counseling, involving the provision of detailed information about TB and the importance of completing the treatment course, was significantly associated with TB medications adherence. Patients are more likely to follow treatment guidelines when they are thoroughly informed, rather than rushed through care in a short time period [33].

The study assessed key socio-demographic, behavioral, and clinical factors by providing a thorough understanding of TB medication non-adherence. Recall bias, where participants may struggle to accurately remember details such as medications use, visit dates, or frequency, could have influenced adherence estimates. To minimize this bias, participants were given adequate time to recall information during interviews, standardized questionnaire was used, and data collectors were well-trained. A pilot survey was also conducted to assess participant experience, helping to further reduce this bias. In addition, reported information was verified using existing medical records wherever possible. Social desirability bias, where participants may provide responses, they perceive as favorable, was minimized through the use of structured interview questions. Consequently, not collecting data from comorbidities like HIV may have introduced residual confounding, as these factors can influence adherence through increased pill burden and potential drug interactions. Future studies could mitigate this limitation by recording and adjusting for these comorbidities in multivariate analysis.

## Conclusion

We found that the prevalence of non-adherence to TB medications among adult patients at the referral hospital was relatively low. Methadone use, spending 1–2 h at the hospital, and having a very good relationship with healthcare workers were associated with non-adherence. Interventions to improve adherence should focus on addressing behavioral factors such as substance use, and enhancing adherence support mechanisms. Furthermore, integrating TB care with substance use services may further improve treatment adherence and improve overall treatment outcomes.

## Supplementary Information

Below is the link to the electronic supplementary material.

Supplementary Material 1

## Data Availability

The datasets during the current study will be available from the corresponding author upon request.

## Declarations

### Authors information

TZ was a postgraduate student pursuing Master of Public Health (MPH) under the supervision of MA and CM, both Senior Lecturers at MUHAS, Tanzania.

## Abbreviations

aPR: adjusted prevalence ratio
CDC: Centre for Disease Control
CI: confidence interval
cPR: crude prevalence ratio
DOT: Directly Observed Therapy
HCWs: Healthcare workers
IQR: interquartile range
MDR-TB: Multi-Drug-Resistant Tuberculosis
MRRH: Mwananyamala Regional Referral Hospital
NSP-IV: National Strategic Plan VI
PCT: Patient Centered TB Treatment
TB: Tuberculosis
WHO: World Health Organization
