# Supplementary material for: Prevalence of TB medication non-adherence and associated factors among adults attending a Regional Referral Hospital in Dar es Salaam, Tanzania
Source: BMC Infect Dis. 2026 Aug 17;26:1980. doi: 10.1186/s12879-026-12886-7 (PMC13757011; doi:10.1186/s12879-026-12886-7)
Supplement: Supplementary file 1 [file 12879_2026_12886_MOESM1_ESM.pdf]

QUESTIONNAIRE:

Part I: Socio-Demographic Characteristics

Participant ID:		
Please give correct answer to the following questions		
1. Age:	--------------------Years	
2. Sex:	1= Male	☐
	2= Female	☐
3.Time in average from home of participant to treatment center.	1=<30 minutes<br>2=>30 minutes	☐<br>☐
4. Education level:	1 = Did not go to school	☐
	2 = Primary education<br>3=Some primary	☐
	4 =Secondary education<br>5=Some secondary	☐
	6 = University education	☐
5. Marital status	1 = Single	☐
	2 = Married	☐
	3 = Divorced	☐
	4 = Widowed	☐
6. Participant employment status	1=Unemployed<br>2=Government employment<br>3=Private employment<br>4=Self-employed.<br>5=Employed but stopped because of TB disease	☐<br>☐<br>☐<br>☐<br>☐

Part II: Health care provider -related factors

1. How long do you spend at the hospital during your visit?	1 = < 1 hour	□
	2 = 1-2 hours	□
	3= 3 hours	□
	4 = > 3 hours	□
3. Are the health care workers usually tell you the importance of taking drugs regularly and adhering to them?	1 = Yes	□
	2 = No	□
5. How can you rate the staffing relationship at the health facility?	1 = Very bad	□
	2 = Bad	□
	3= Indifferently	□
	4 = Good<br>5=Very good	□<br>□

Part III Patient-Related Factor

(A). WHO Alcohol UseDisorder Identification Test (AUDIT))

Participant ID	
**The Alcohol Use Disorders Identification Test: Interview Version**<br>Read questions as written. Record answers carefully. Begin the AUDIT by saying "Now I am going to ask you some questions about your use of alcoholic beverages during this past year." Explain what is meant by "alcoholic beverages" by using local examples of local f beer, wine, vodka, etc. Code answers in terms of "standard drinks". Place the correct answer number in the box at the right.	
1. How often do you have a drink containing alcohol?<br>(0) Never [Skip to Qs 9-10]<br>(1) Monthly or less<br>(2) 2 to 4 times a month<br>(3) 2 to 3 times a week<br>(4) 4 or more times a week<br>☐	6. How often during the last year have you needed a first drink in the morning to get yourself going after a heavy drinking session?<br>(0) Never<br>(1) Less than monthly<br>(2) Monthly<br>(3) Weekly<br>(4) Daily or almost daily<br>☐
2. How many drinks containing alcohol do you have on a typical day when you are drinking?<br>(0) 1 or 2<br>(1) 3 or 4<br>(2) 5 or 6<br>(3) 7, 8, or 9<br>(4) 10 or more<br>☐	7. How often during the last year have you had a feeling of guilt or remorse after drinking?<br>(0) Never<br>(1) Less than monthly<br>(2) Monthly<br>(3) Weekly<br>(4) Daily or almost daily<br>☐

3. How often do you have six or more drinks on one occasion?<br>(0) Never<br>(1) Less than monthly<br>(2) Monthly<br>(3) Weekly<br>(4) Daily or almost daily<br>Skip to Questions 9 and 10 if Total Score for Questions 2 and 3 = 0<br>☐	8. How often during the last year have you been unable to remember what happened the night before because you had been drinking?<br>(0) Never<br>(1) Less than monthly<br>(2) Monthly<br>(3) Weekly<br>(4) Daily or almost daily<br>☐
4. How often during the last year have you found that you were not able to stop drinking once you had started?<br>(0) Never<br>(1) Less than monthly<br>(2) Monthly<br>(3) Weekly<br>(4) Daily or almost daily<br>☐	9. Have you or someone else been injured as a result of your drinking?<br>(0) No<br>(2) Yes, but not in the last year<br>(4) Yes, during the last year<br>☐
5. How often during the last year have you failed to do what was normally expected from you because of drinking?<br>(0) Never<br>(1) Less than monthly<br>(2) Monthly<br>(3) Weekly<br>(4) Daily or almost daily<br>☐	10. Has a relative or friend or a doctor or another health worker been concerned about your drinking or suggested you cut down?<br>(0) No<br>(2) Yes, but not in the last year<br>(4) Yes, during the last year<br>☐
Sum up specific items values to calculate total, then record here ☐	
NB: If total is greater than recommended cut-off, consult User's Manual	

(B). Tobacco assessment questionnaire

Participant ID:		
Please ask the participant, the following question and tick for each question in the provided box		
1. Do you currently use one or more of the following tobacco products (cigarettes, snuff, chewing tobacco, cigars, hookah (shisha), etc.)?	1 = Ye:	☐
	2 = No:	☐
2. In the past month, how often have you used one or more of the following tobacco products (cigarettes, snuff, chewing tobacco, cigars, hookah(shisha) ,etc.)?	1=Once ortwice	☐
	2=Weekly	☐
	3= Almostdaily	☐
	4 = Daily	☐
3. How soon after waking up do you smoke your first cigarette?	0 = 60+ minutes	☐
	1 = 31-60 minutes	☐
	2 = 5-30 minutes	☐
	3=Within 5 minutes	☐
4.Do you currently live with someone who smokes?	1 = Ye:	☐
	2 = No:	☐
5. How many cigarettes a day do you smoke?	0 = 10 or less	☐
	1 = 11 – 20	☐
	2 = 21 – 30	☐
	3= 31 ormore	☐

(C). Family support and traditional healers

3.How the family member is supportive for your treatment?	1 = Never	☐
	2 = Somehow	☐
	3= Always	☐
4.Are you using any traditional medicines to treat TB disease?	1 = Yes	☐
	2 = No	☐

Part IV: Medicine -Related Factors

1(a). Do you experience any side effect when taking TB medications?	1 = Yes	☐
	2 = No	☐
1(b). If the answer in 2(a) above is yes, which side effects?	1 = Diarrhea and vomiting	☐
	2 = Skin rashes	☐
	3= Headache and Dizziness<br>4=Numbness<br>5=Yellow coloration of eyes<br>6=Others	☐<br>☐<br>☐<br>☐
2. Are you always tolerating taking the TB tablets without any problem?	1=Yes	☐
	2=No	☐
		☐
		☐

3(a)Are you taking other medications apart from TB medications?	1 = Ye	☐
	2 = No	☐
(b). If yes to the above question, which medications are you takings?	1 =Antiretroviral therapy	☐
	2 = Methadone	☐
	3 = Anti-hypertension	☐
	4 = Diabetes medications<br>5 = Others	☐<br>☐

Part V: Condition-Related factors

LIST OF COMORBIDITIES		
Please ask the participant following question and tick the response in the box provided		
**1.**HIV	1 = Reactive<br>2 = Non-reactive	□<br>□
2.Diabetes Mellitus	1=Diabetic<br>2=Non-diabetic	□<br>□
3.Hypertension	1=Hypertensive<br>2=Not-Hypertensive	□<br>□
4.Drug Abuse	1= Drug abuser<br>2= Non-drug abuser	□<br>□
5.Other	1=Yes<br>2=No<br>If Yes(mention)_________	□<br>□

Part VI: Self-report questionnaire

Adherence to TB medications measurement

**Participant ID_**	
**Date of treatment initiation**	*I__I__I__I__I__I__I__I__I*

Begin the questionnaire by saying *"Most people with TB have many pills to take at different times during the day. Many people find it hard to always remember to take their pills. It is important for me to understand how you are really doing with your medicine. Don't worry about telling me if you don't always take all your doses. I need to know what is really happening, not what you think I want to hear."*

Question	Yes	No
1. Do you sometimes find it difficult to remember to take your medication?	☐	☐
2. When you feel better, do you sometimes take a break from your medication?	☐	☐
3. Thinking back over the past four days, have you missed any of your doses?	☐	☐
4. Sometimes if you feel worse when you take the medicine, do you stop taking it?	☐	☐

If all 4 answers are **No,** then the patient is classified as being adherent.

____________________ ____________ _______________ _______________

Name of the interviewer Title Signature Date
